# COVID-19 Pandemic and Teachers’ Classroom Safety Perception, Anxiety and Coping Strategies during Instructional Delivery

**DOI:** 10.3390/healthcare10050920

**Published:** 2022-05-17

**Authors:** Frank Quansah, James Boadu Frimpong, Francis Sambah, Prince Oduro, Stephen Kofi Anin, Medina Srem-Sai, John Elvis Hagan, Thomas Schack

**Affiliations:** 1 Department of Educational Foundations, University of Education, Winneba P.O. Box 25, Ghana; fquansah@uew.edu.gh; 2 Department of Health, Physical Education and Recreation, University of Cape Coast, PMB TF0494, Cape Coast P.O. Box 5007, Ghana; james.frimpong@stu.ucc.edu.gh (J.B.F.); francis.sambah@stu.ucc.edu.gh (F.S.); 3 Department of Special Education, University of Education, Winneba P.O. Box 25, Ghana; poduro@uew.edu.gh; 4 Department of Industrial and Health Sciences, Faculty of Applied Sciences, Takoradi Technical University, Takoradi P.O. Box 256, Ghana; stephen.anin@ttu.edu.gh; 5 Department of Health, Physical Education, Recreation and Sports, University of Education, Winneba P.O. Box 25, Ghana; mssai@uew.edu.gh; 6 Neurocognition and Action-Biomechanics-Research Group, Faculty of Psychology and Sports Science, Bielefeld University, Postfach 10 01 31, 33501 Bielefeld, Germany; thomas.schack@uni-bielefeld.de

**Keywords:** anxiety, coping mechanisms, COVID-19, instructional delivery, safety perception, teachers

## Abstract

Several professionals, including teachers, have been engrossed in fear of the worst happening due to COVID-19 and the rapidly evolving nature of the virus affecting the perception of safety in any working environment. This study examined teachers’ perception of classroom safety, anxiety, and coping strategies during instructional delivery in senior high schools in Ghana. Adopting the cross-sectional survey design with a quantitative approach, a convenient sample of 174 senior high school teachers in the Cape Coast Metropolis completed a questionnaire. Data were analyzed using descriptive analysis, analysis of variance, and multiple linear regression. The findings showed that teachers perceived their classroom environment as unsafe during instructional delivery amidst COVID-19 and reported modest to extreme levels of anxiety. Further, teachers with a high level of COVID-19 anxiety were found to utilize active coping strategies to manage stressful situations. The reported unsafe working environment in schools during pandemic times highlights the critical role of supportive working environments for teachers’ mental and psychological wellness. School counseling psychologists, school welfare officers, and school health coordinators should collaborate to implement interventions (e.g., social emotional learning; resilience strategies) that promote the wellbeing of teachers and a safe working environment.

## 1. Introduction

The outbreak of the COVID-19 pandemic has undoubtedly disrupted many aspects of human lives and activities all over the world. Essentially, the pandemic has greatly affected and exposed the strengths and weaknesses in the public health systems in many countries worldwide, with the education sector having its fair share through a spillover effect [1,2]. The United Nations Educational Scientific and Cultural Organization (UNESCO) reported that the education sector is among the most disrupted fields affecting approximately 1.6 billion students worldwide in more than 190 countries [3]. The pandemic’s multifaceted impact on classroom teachers’ and students’ health, and students’ learning at all levels of the educational systems across the world is unprecedented [4,5,6]. As a measure to reduce the incessant spread of the virus, governments and world leaders shut down schools and universities since the closure of educational institutions was considered a significant tool for enhancing social and physical distancing among students and teachers [7].

Several categories of professionals experienced fear and have been concerned about their safety in the COVID-19 era. For example, student teachers [8,9], tourism and construction workers [10,11,12], healthcare professionals [13,14], and teachers [15,16,17] have expressed concern about their safety during the COVID-19 outbreak. Particularly in Ghana, Quansah et al. [8] revealed that physical education teachers perceived the teaching environment as largely unsafe, increasing their level of anxiety and fear. Similarly, Hagan et al. [9] surveyed the general teacher population in selected senior high schools in Ghana and found evidence of anxiety prevailing among the teachers. Admittedly, being preoccupied with extreme concerns about one’s safety within one’s working environment may affect overall productivity [18,19]. Classroom safety perception refers to the views held by persons who operate within a pedagogical setting about the level of risks or potential hazards they might be exposed to. Within the classroom environment, the COVID-19 effect has negatively impacted teaching and learning, and this is further compounded by the scarcity of resources such as teaching and learning materials and equipment, personal protective equipment (PPEs), facilities, and other logistics, particularly in developing countries such as Ghana [20,21]. Research has shown that these challenges lead to heightened fear of COVID-19 and increase the level of anxiety of teachers regarding their safety in the school setting [22].

However, since the pandemic seems to linger on, teachers need to devise mechanisms to cope with the physical and psychological reactions such as anxiety and stress that accompany the pandemic. Available research indicates that teachers have made several adjustments in response to the pandemic, such as switching to digital and online learning [23,24,25] and adopting psychological coping strategies [26,27]. For instance, Klapproth et al. [27] found that even though most of the teachers in Germany experienced COVID-19 adversities, they coped functionally with them. Coping strategies refer to behavioral and cognitive strategies used by persons to manage crises, conditions, and demands that are perceived as distressing [28,29,30]. These strategies can be classified into four, namely cognitive approach, cognitive avoidance, behavioral approach, and behavioral avoidance [29,31].

Drawing from Maslow’s [32] hierarchy of needs, safety is a fundamental need for all humans that ought to be satisfied before one can enjoy other needs such as the feeling of love and attachment to others, feeling good about one’s achievement, and achieving one’s full potential. Essentially, personal safety is realized when an individual can completely function with little or no imaginary perceptions or actual experiences of physical or mental torture [33]. Hence, creating a safe teaching atmosphere for teachers by providing safety plans, connectedness, and support systems could help them discharge their duties diligently and be pleased with their teaching positions [34].

Despite the heightened physical and psychological (stress and anxiety) burden of COVID-19 across different populations [35,36,37,38,39,40,41], it is surprising that studies assessing Ghanaian teachers’ perception of the safety of the classroom environment, their level of anxiety, coping strategies and the possible relationship between anxiety and coping strategies adopted during the COVID-19 era remain untapped. This creates a knowledge gap in mental health and psychology literature which the current study seeks to address. The study could influence policymakers to develop and implement interventions to improve teacher safety in the teaching-learning environment and preserve their overall physical and mental health.

Therefore, the study aimed to examine teachers’ perception of classroom safety, anxiety, and coping strategies during instructional delivery in senior high schools in Ghana. Four questions guided the conduct of this study: (1) what are teacher perceptions regarding the safety of the classroom environment during teaching, (2) what is the extent of anxiousness exhibited by teachers towards contracting COVID-19 during instructional delivery, (3) what are the adopted coping strategies of teachers during instructional delivery amidst the COVID-19 pandemic and (4) how does COVID-19 related anxiety of teachers influence their coping strategies during the COVID-19 pandemic? The study covered senior high school teachers, who are instructors at the secondary school level. The decision to use teachers at the secondary school level was made because teachers at the other levels of education (e.g., basic education level) had vacated and were not available at the time of the study. Moreover, previous studies have provided evidence of fear and anxiety among teachers at the secondary school level [8,9].

## 2. Materials and Methods

### 2.1. Research Approach and Design

This study employed the quantitative research approach through a cross-sectional design to address teacher perceptions of classroom safety and anxiety and the coping strategies used by senior high school teachers during instructional delivery amidst the COVID-19 pandemic. Thus, owing to the main question, this approach was appropriate as it draws on yielding valuable knowledge and understanding of the issues under investigation using statistical procedures by collecting data from teachers during the COVID-19 period [42]. In recent times, this approach and design have been supported by scholars who have researched similar issues [12,13]. Using a survey method was advantageous and considered suitable because it was possible to recruit several participants more conveniently, even amid the COVID-19 constraints.

### 2.2. Sampling

The study participants comprised 174 senior high school teachers in the Cape Coast Metropolis, Central Region, Ghana, with 118 males (67.8%) and 56 females (32.2%). The youngest respondent was 20 years old, while the oldest was 52 years, with a mean age of 36 years. Nearly 11.5% of the respondents had worked less than 1 year, 31.0% had worked between 1–2 years, 55.2% had worked between 3–4 years, and 44.8% had worked for more than 5 years. The participants were selected through the convenience sampling technique. The study was carried out when only the final year students were in school preparing for their examinations. Consequently, most of the participants were final-year teachers, with very few first and second-year teachers who were randomly found on the premises during the data collection. Every senior high school teacher within the Cape Coast Metropolis qualified to take part in the study. The choice of the metropolis was influenced by the researchers’ experiences with the teachers during the supervision exercise. That is, we observed that teachers within the metropolis exhibited some level of fear which appeared to have affected their interaction in the school. This was further supported by findings from previous studies conducted within the same metropolis [8,9].

### 2.3. Measurement of Variables

#### 2.3.1. Teachers’ Perception of Classroom Safety

In the context of this study, classroom safety was conceptualized as the degree to which teachers perceived the teaching environment as safe in terms of contracting COVID-19 in their line of duty. Five items were adopted from Capone et al.’s [43] standardized measure of perceived environmental safety during COVID-19. These items served as proxies to measure the teachers’ perception of classroom safety. Some of the statements posed to the participants were: “*I fear contracting COVID-19 when teaching the students*”, “*I feel too exposed to COVID-19 during instructional delivery*” and “*I feel susceptible to contracting COVID-19 in the classroom when teaching.*” The participants responded to the 5-items using “*Yes*” or “*No*” response options. The internal consistency for the items was computed using the Kuder–Richardson 21 reliability estimate [44,45,46,47], which yielded an estimate of 0.73.

#### 2.3.2. COVID-19 Anxiety

COVID-19 anxiety reflects the extent of nervousness experienced by the participants due to the fear of contracting the COVID-19 virus during instructional delivery. The anxiety scale developed and calibrated by Beck et al. [48] was adopted as the measure for the anxiety variable. In adapting the anxiety scale, only items on the non-clinical symptoms of anxiety were selected for use. This is because the participants in this study were not ‘clinical patients’ (i.e., people diagnosed with a particular condition and/or hospitalized in a health facility. These items were measured using a scale option of 0–3 (i.e., 0 representing “*not at all*”, 1 indicating “*somewhat*”, 2 depicting “*moderately*”, and 3 representing “*very much so*”). The participants were required to indicate the extent to which they experienced some listed symptoms. The 6 items included: “*I fear the worst happening*”, “*I feel nervous*”, “*I feel unrelaxed*”, “*I feel unsteady*”, “*I feel very much concerned*”, and “*I have self-doubt*”. The reliability analysis was performed with Omega ω [49,50,51,52], which yielded an estimate of 0.76. The Beck’s anxiety scale was adapted for this study because the scale is the oldest, most popular, and well-accepted measure of anxiety compared to other anxiety measures like the Coronavirus Anxiety Scale [48]. Thus, several validation studies have been conducted on the scale across diverse cultural boundaries (including Ghana), widening its sensitivity, applicability, and generalization.

#### 2.3.3. Coping Strategies

A well-developed and calibrated multi-dimensional cultural-mix coping inventory (i.e., Cultural-mix Inventory for Stressful Situations Involving Coping Mechanisms) was adopted as the coping measure for this study [53]. This coping inventory was utilized for this study due to two reasons: (1) it is the only coping inventory developed in Ghana, and (2) the inventory happens to be among the few coping measures which have a mix of religion and emotional support together with other conventional coping strategies. Although the coping measure was initially developed for students, it has also been validated among teachers. Moreover, the coping inventory is very flexible since the items are not very specific to a particular context. Hence, it is accommodating to other populations [53]. The coping inventory has four dimensions with 4-items each. The sub-dimensions include active coping (e.g., “*I concentrate my effort on doing something* *about it*”, “*I take additional action to try to get rid of the problem*”), religious coping (e.g., “*I put my trust in God/object of worship*”, “*I pray more than usual for my God to guard me*”), behavior disengagement coping (e.g., “*I just give up trying to reach my goal because of the stressor*”, “*I reduce the amount of effort I’m putting into solving the problem*”) and social support (e.g., “*I discuss how I feel about the stressor with someone*”, “*I get sympathy and understanding from someone to reduce my fears about the problem*”). The Omega ω reliability coefficients for the active coping, religious coping, behavior disengagement and the emotional support dimensions are 0.82, 0.81, 0.87 and 0.83, respectively.

### 2.4. Data Collection Procedure and Ethical Considerations

Prior to data collection, permission was sought from headmasters of all senior high schools in the Cape Coast Metropolis to allow their teaching staff to participate in the study. This was after an ethical clearance had been issued by the Institutional Review Board of the University of Cape Coast in Ghana, with reference number UCCIRB/EXT/2020/25. Participants who showed interest in taking part in the study were debriefed accordingly in their various schools. Debriefing was organized in two sessions, individually and in groups. The debriefing session tackled issues of confidentiality, respect for the rights of the study participants and withdrawal from the research project without any legal bindings. Full disclosure of the nature of the study, including general and specific objectives, and the emotional risk associated with the study was explained to the participants before questionnaire administration. Consent forms were duly signed by all participants. Two hundred questionnaires were distributed to the teachers to respond to. Under the same conditions, the participants were required to complete the questionnaires accurately and as honestly as possible without any influence from others. The questionnaire administration lasted for two months. The participants were informed not to write their names on the questionnaires during the data collection, assuring them that their data would remain anonymous. The data were obtained on-site, in the staff room of the teachers. Teachers who opted for another place to respond to the instruments were allowed to do so. Out of 200 questionnaires distributed, 174 were completed and returned leading to a response rate of 87%. Fifteen (15) out of the 200 questionnaires had over 80% of missing responses and were considered incomplete. Eleven (11) other teachers opted out of the study. The remaining valid 174 answered questionnaires were processed for analysis. The investigators protected the psychological wellbeing of all participants [54].

### 2.5. Data Analysis Plan

The data were analyzed using both descriptive and inferential analysis. Frequencies and percentages were used to represent participants’ responses on their perceptions regarding the safety of the classroom environment during teaching amid the COVID-19 pandemic. To establish the extent of anxiety experienced by the participants towards contracting COVID-19 during instructional delivery, the mean values were used to classify participants as experiencing a mild, moderate or extreme level of anxiety using Beck et al.’s [48] criteria. Mean scores and standard deviation were used to rank the extent to which coping strategies were adopted by teachers when teaching during the COVID-19 pandemic. Further, repeated measures analysis of variance (ANOVA) was performed to assess the extent to which the teachers differed in using the coping strategies. Multivariate multiple linear regression (MMLR) analysis was also conducted to assess the influence of anxiety on the coping strategies of teachers during the COVID-19 pandemic. For the MMLR analysis, a Bonferroni adjustment was used to reduce type 1 error, and as such, an alpha level of 0.0125 was used instead of the usual 0.050. Before the MMLR analysis, assumptions such as multivariate normality and outliers were satisfied [55].

## 3. Results

### 3.1. Teachers’ Perception Regarding the Safety of Classroom Environment during Teaching

The study explored the perception of teachers regarding the safety of the classroom environment amidst the COVID-19 pandemic. The frequency and percentage distribution of the participant responses are shown in Table 1.

As presented in Table 1, it was revealed that the teachers feared getting infected with COVID-19, felt exposed and susceptible to the virus, and felt uneasy. The general impression from the results depicts that the teachers perceived the classroom environment as largely unsafe in terms of the possibility of contracting the virus within the teaching and learning settings.

### 3.2. The Anxiety Experiences of Teachers towards Contracting COVID-19 during Instructional Delivery

The research further assessed the extent of anxiety experienced by teachers towards contracting COVID-19 during instructional delivery. The participants were assigned a specific level of anxiety they experienced based on their responses to the items. The categorization provided by Beck et al. [48] was adopted in this study to classify participants into three groups: those experiencing mild anxiety, moderate anxiety, and extreme anxiety. Participants with mean scores ranging between 0–1.4 were considered as experiencing mild anxiety, those with mean scores larger than 1.4 but less than a score of 2.5 were considered as experiencing moderate anxiety, and those with mean values of 2.5 or more were classified as exhibiting extreme anxiety. The levels of anxiety are shown in Table 2.

The results revealed that the teachers experienced modest to extreme levels of anxiety. Particularly, over 50% of the teachers reported a moderate level of anxiety (see Table 2). Quite a large number of the teachers showed an extreme level of anxiety (45.4%).

### 3.3. Coping Strategies of Teachers during Instructional Delivery Amidst COVID-19 Pandemic

The participants were required to indicate coping strategies they adopted during the instructional delivery amid the COVID-19 pandemic. This objective also examined whether the teachers significantly differed in adopting each coping strategy. The details of the results are presented in Table 3.

The results showed that the extent to which the teachers used the coping strategies differed from one type of coping strategy to another, *F*(3, 17) = 99.36, *p* < 0.001 (see Table 3). Using the Sidak multiple comparison approach, all paired comparisons showed a significant result, except for emotional support and active coping. For example, the extent to which the teachers adopted a religious coping strategy was significantly different from the extent to which emotional support coping was adopted (*p* < 0.001). Similarly, the extent to which the teachers utilized active coping was different from the degree to which behavior disengagement coping was adopted (*p* < 0.001). Given this, the study showed that teachers mainly used the religious coping strategy (*M* = 3.23, *SD* = 0.76) to cope with the stressful situation presented by the COVID-19 pandemic. This was followed by emotional support coping (*M* = 2.64, *SD* = 0.66), then active coping (*M* = 2.59, *SD* = 0.64), and finally the behavior disengagement coping (*M* = 1.77, *SD* = 0.75).

### 3.4. Influence of Anxiety of Teachers on Their Coping Strategies during the COVID-19 Pandemic

The researchers further examined how the extent of COVID-19 anxiety influenced teacher coping strategies during the COVID-19 pandemic. The results from the multivariate linear regression analysis are shown in Table 4.

As shown in Table 4, the analysis revealed that COVID-19 related anxiety positively predicted active coping strategy, *B* = 0.212, *SE* = 0.092, *p* = 0.012. Further, COVID-19 anxiety was found to be negatively related to emotional support, *B* = −0.204, *SE* = 0.094, *p* = 0.013. Anxiety was not found to be significantly associated with behavior disengagement and religious coping strategies.

## 4. Discussion

This study explored teachers’ perception of the safety of the classroom teaching environment amidst the COVID-19 pandemic, anxiety about contracting the virus during instructional delivery, and the coping strategies adopted. The study findings showed that teachers perceived the classroom environment as unsafe during instructional delivery amidst COVID-19. For instance, the teachers reported fear of contracting the virus during teaching, uneasiness when interacting with students, and feeling exposed and susceptible to COVID-19 when teaching in the classroom. This concurs with findings from similar studies where teachers [15,16,17], student teachers [8,9], tourism and construction workers [10,11,12], and healthcare professionals [13,14] expressed concerns about their safety within their work environment during the COVID-19 outbreak. This finding has far-reaching consequences on teaching and learning. A previous study has established that a safe school environment is critical to optimizing learning as it protects learners, teachers, and staff and engenders trust among stakeholders [56]. However, the pervasion of the COVID-19 pandemic has altered the safety of so many working environments, including the schools where teachers work. This is affirmed by reports from teachers regarding the fear of contracting the virus during teaching, uneasiness when interacting with students, and feeling exposed and susceptible to COVID-19 when teaching in the classroom. When teachers do not feel safe, the learning environment is compromised, leading to subpar instruction, poor teacher-student relationships, and high drop-out rates among teachers [57]. The teachers’ perception of their safety may be due to the lack of PPEs [58] that sometimes characterized the response to the pandemic in Ghana, coupled with the lack of knowledge about the scientific preventive protocols that work to enhance protection [59]. Teacher burnout, a psychological syndrome that involves a prolonged response to stressors in the workplace, was reported to be associated more with the lack of resources (preparation and information) for alternative teaching methods such as the use of digital media and online resources for teachers, and not necessarily the extra workload that came along with the pandemic [60,61]. Stakeholders in education service delivery should endeavor to provide and maintain a safe school environment to incentivize work output [62].

Further, the study showed that most of the teachers reported modest to extreme levels of anxiety during instructional delivery amidst COVID-19. This is much expected as the unpredictability, limited understanding, and novel character of the COVID-19 brought uncertainties with alarming morbidities and mortalities reported with most healthcare resources and facilities overstretched. Learning is a social process where instructors and learners mostly congregate. These meetings predispose teachers and learners alike to disease pathogens like COVID-19 and stress, and if adequate measures are lacking, they may endanger safety [63]. This is similar to the findings of a Ghanaian study by Quansah et al. [9], where student teachers reported moderate to high anxiety during instructional delivery amidst COVID-19. The observed similarity explains the unique and widespread fear and panic brought about by the COVID-19 among humanity. This, among other factors, might have informed most educational institutions’ decision to migrate teaching and learning to e-platforms [23,24,25].

In order to attenuate the impact of the pandemic, many have adopted various coping mechanisms that seem to work and enhance the quality of life amidst the pandemic. This was also revealed in our study, where teachers predominantly reported the use of religious coping, emotional support, and active coping to help minimize the negative anxiety associated with the pandemic nuances. Besides, our study also found that behavior disengagement was minimally adopted by the teachers when coping with COVID-19 anxiety during teaching. These findings agree with Klapproth et al.’s [27] study, where teachers predominantly adopted functional coping strategies such as active, religious, and emotional coping and minimally utilized the dysfunctional coping mechanisms such as behavior disengagement to manage COVID-19 related stress. Likewise, Erschens et al.’s [64] study among medical students revealed the use of both functional and dysfunctional coping to manage burnout situations. The fact that teachers predominantly use functional coping strategies shows their deliberate attempt to deal with and manage their COVID-19 anxiety, although minimal behavior disengagement coping was also applied.

This study also showed that teachers with a high level of COVID-19 anxiety were found to utilize active coping strategies in dealing with a stressful event. This finding is encouraging because teachers probably saw themselves as active participants in their environment and that they had a contribution to make to alter the negative impact of the pandemic. Such positive adaptive coping strategies will be significantly profiting due to the possible long-term coexistence of the COVID-19 pandemic. The teachers with high anxiety levels utilized more active coping probably due to their sense of control [65]. For example, teachers who have a high sense of control of the COVID-19 situation even in the presence of high anxiety will be more likely to adopt active coping strategies. Such teachers will put in efforts (such as social distancing, washing of hands, and consistent use of hand sanitizer) towards preventing the infection of the virus. This explains why the study revealed that such teachers utilized low emotional support coping. Therefore, it is necessary that educational institutions put in interventions that would aim to improve teachers’ adaptive coping resilience to promote wellbeing, as stressed by Wang et al. [66].

The study showed that behavior disengagement and religious coping strategies were not significantly associated with COVID-19 anxiety. In other words, the teachers’ level of COVID-19 anxiety did not determine whether they would adopt behavior disengagement and religious coping strategies in dealing with stressful events. Perhaps, the teachers preferred and adopted other coping mechanisms that they perceived or believed to be more functional in suppressing the debilitative psychological health outcomes such as anxiety and panic [67,68].

### 4.1. Strengths and Limitations

This study contributes significantly to understanding the workplace safety of teachers during instructional delivery amidst the COVID-19 pandemic within the Ghanaian context and other similar settings in developing countries. Stakeholders and policymakers of secondary school education may now better appreciate the arduous task of teachers since most teaching and learning in Ghanaian senior high schools employ predominantly traditional face-to-face methods where everyone gathers at a confined place for lesson delivery. These findings, therefore, provide an opportunity for stakeholder reflection on the safety of both teachers and learners during infectious pandemics to create a congenial and safe climate for teachers and learners. The study, therefore, provides evidence of the intangible costs (e.g., stress, anxiety, pain) associated with an unsafe workplace climate that teachers are exposed to during this pandemic period. This study also adds to the sparse literature on teachers’ classroom safety amidst the pandemic. Despite these strengths, our study is not without limitations. Foremost, the use of convenient sampling to recruit participants from one geographic region limits the representativeness and generalizability of findings to all teachers in Ghana. Secondly, the cross-sectional nature of the study design precludes causal inferences of any sort. No interaction analysis was conducted, so the potential mediating effect of some variables may have been overlooked in this study. Future research should use longitudinaldesigns to establish trendss of teachers’ anxiety experiences during instructional delivery among a larger teacher population during this lingering pandemic.

### 4.2. Practical Implications

The present findings have some useful, practical implications for the teaching industry in Ghana since humanity may continue to encounter this pandemic for the foreseeable future. The reported unsafe working environment in schools during pandemic times highlights the critical role of supportive working environments for teachers’ mental and psychological wellness. This brings to focus the role of school counseling psychologists, school welfare officers, and school health coordinators in collaborating for the better wellbeing of teachers. Moreover, the reported anxiety induced by the perceived unsafe working environment implies that teacher absenteeism, learner attrition, and poor learning outcomes could increase. The study also points to the need for education authorities to develop innovative learning modules that embrace technology and upgrade the technological prowess of teachers to save teaching jobs. Additionally, the findings highlight the usefulness of functional coping strategies, which are encouraging and should be enhanced for teachers’ mental resilience and wellbeing during the ongoing pandemic.

## 5. Conclusions and Recommendations

The present findings stress the need for the safety of teachers in their workplace amidst COVID-19 since the extent of safety influences their anxiety level and further determines the choice of coping strategies. The study concludes that the COVID-19 pandemic resulted in moderate to high anxiety among teachers, particularly because they felt unsafe about the teaching and learning environment. The study’s findings suggest that stakeholders in education service delivery should endeavor to provide and maintain a safe school environment as this would incentivize work output. Moreover, insurance cover should be provided for teachers who get infected during such disease outbreaks in the line of work. This approach will motivate teachers to put up their best to optimize learning outcomes. Further, the Central regional education directorate of the Ghana Education Service should consider enhanced teacher training and integration of ICT into the teaching industry. This could mitigate teacher anxiety while teaching and learning activities are migrated unto online platforms exclusively or to complement the traditional in-person teaching methods. Educational institutions are encouraged to promote interventions (e.g., social emotional learning) that aim to improve teachers’ resilience to promote their wellbeing and facilitate effective instructional delivery.

## Figures and Tables

**Table 1:** Teacher responses on their perception of classroom safety during teaching.

No.	Statements	No, *n* (%)	Yes, *n* (%)
1.	I fear contracting COVID-19 when teaching the students	73 (42.0)	101 (58.0)
2.	I feel too exposed to COVID-19 during instructional delivery	71 (40.8)	103 (59.2)
3.	I am uneasy when interacting with the students amidst COVID-19	59 (33.9)	115 (66.1)
4.	I feel susceptible to contracting COVID-19 in the classroom when teaching	22 (12.6)	152 (87.4)
5.	The classroom environment is not protective enough during teaching	69 (39.7)	105 (60.3)

**Table 2:** Frequency distribution of teachers’ level of anxiety.

Anxiety Level	Frequency	Percent	Mean	SD
Mild	3	1.7	1.74	0.53
Moderate	92	52.9		
Extreme	79	45.4		

**Table 3:** Coping strategies adopted by teachers when teaching during the COVID-19 pandemic.

Coping Strategies	Mean	SD	Rank	Repeated Measures ANOVA			
				*F*	*df1*	*df2*	*p*
Religious coping	3.23	0.76	1st	99.36 *	3	171	0.000
Emotional support coping	2.64	0.66	2nd				
Active coping	2.59	0.64	3rd				
Behaviour disengagement	1.77	0.75	4th				

* *F*-value significant at *p* < 0.001.

**Table 4:** Parameter estimates for the prediction of anxiety on coping strategies adopted by teachers.

Dependent Variable	Parameter	*B*	Std. Error	*t*	*p*	95% Confidence Interval	
						Lower Bound	Upper Bound
Active Coping	Intercept	2.221	0.167	13.298	0.000	1.891	2.550
	Anxiety	0.212	0.092	2.304	0.012 *	0.030	0.394
Religious Coping	Intercept	2.959	0.199	14.879	0.000	2.567	3.352
	Anxiety	0.157	0.110	1.429	0.155	−0.060	0.373
Behavior disengagement	Intercept	1.613	0.198	8.136	0.000	1.222	2.005
	Anxiety	0.092	0.109	0.841	0.402	−0.124	0.308
Emotional Support	Intercept	2.992	0.171	17.525	0.000	2.655	3.328
	Anxiety	−0.204	0.094	−2.164	0.013 *	−0.389	−0.018

* significant at *p* ≤ 0.013.

## Data Availability

The data are available upon reasonable request through the corresponding author.
